# Development of a hybrid bio-purification process of lactic acid solutions employing an engineered *E. coli* strain in a membrane bioreactor

**DOI:** 10.1186/s13068-024-02497-2

**Published:** 2024-03-30

**Authors:** Alexandra Nastouli, Joseph Sweeney, Michael Harasek, Anastasios J. Karabelas, Sotiris I. Patsios

**Affiliations:** 1 Laboratory of Natural Resources and Renewable Energies, Chemical Process and Energy Resources Institute (CPERI), Centre for Research and Technology-Hellas (CERTH), Thermi, Thessaloniki, Greece; 2 Institute of Chemical, Environmental and Bioscience Engineering, TU Wien, Vienna, Austria; 3 School of Biosystems and Food Engineering, University College Dublin (UCD), Belfield, Dublin, Ireland

**Keywords:** Bio-purification, Lactic acid, Membrane bioreactor, *E. coli*, Selective catabolism, Fermentation, Ultrafiltration, Biotechnology, Downstream processing

## Abstract

**Background:**

A potential alternative to lactic acid production through sugar fermentation is its recovery from grass silage leachate. The separation and purification of lactic acid from fermentation broths remain a key issue, as it amounts to up to 80% of its industrial production cost. In this study, a genetically engineered *E. coli* strain (A1:ldhA), that cannot catabolize lactic acid, has been used to selectively remove impurities from a synthetic medium comprising typical components (i.e., glucose and acetic acid) of green grass silage leachate. A systematic approach has been followed to provide a *proof-of-concept* for a bio-purification process of lactic acid solutions in a membrane bioreactor operating in semi-continuous mode.

**Results:**

The synthetic medium composition was initially optimized in shake-flasks experiments, followed by scale-up in bench-scale bioreactor. Complete (i.e., 100%) and 60.4% removal for glucose and acetic acid, respectively, has been achieved in batch bioreactor experiments with a synthetic medium comprising 0.5 g/L glucose and 0.5 g/L acetic acid as carbon sources, and 10 g/L lactic acid; no lactic acid catabolism was observed in all batch fermentation tests. Afterwards, a hybrid biotechnological process combining semi-continuous bioreactor fermentation and ultrafiltration membrane separation (membrane bioreactor) was applied to in-situ separate purified medium from the active cells. The process was assessed under different semi-continuous operating conditions, resulting in a bacteria-free effluent and 100% glucose and acetic acid depletion, with no lactic acid catabolism, thus increasing the purity of the synthetic lactic acid solution.

**Conclusions:**

The study clearly demonstrated that a bio-purification process for lactic acid employing the engineered *E. coli* strain cultivated in a membrane bioreactor is a technically feasible concept, paving the way for further technological advancement.

**Supplementary Information:**

The online version contains supplementary material available at 10.1186/s13068-024-02497-2.

## Background

Lactic acid (LA) is a biomolecule with two optical isomers in nature, D- and L-LA. It is widely used in industry, i.e., in medical and pharmaceutical, cosmetic, food, polymer and textile sector [1, 2]. Although, LA can be produced by a chemical synthesis process, 90% of the LA worldwide demand is produced through sugars’ fermentation [3]. The specifications of LA purity level depend on its industrial use, i.e., pharmaceutical industry requires higher than 90% purity, while food-grade LA has 80–90% purity [2]. Due to the physicochemical properties of LA, such as high water affinity [1], as well as the wide range of impurities in the fermentation broth (e.g., residual sugars, heavy metals and organic acids), downstream purification remains a significant bottleneck of the LA production process through fermentation [4]. Specifically, it has been estimated that LA purification may comprise between 30% and 40% [5] and 80% [6] of the total LA production cost, depending on the targeted purity level. Thus, the efficiency of the downstream purification process is a key issue that research has focused on. [2].

Currently, LA produced from sugars’ fermentation at industrial scale requires a series of downstream processing steps of the fermentation broth and usually include: cells’ separation, precipitation in the form of slightly soluble lactate salts, e.g., by addition of Ca(OH)_2_, filtration, acidification, carbon adsorption, evaporation, and crystallization process [1]. Precipitation using Ca(OH)_2_ usually requires an excess of H_2_SO_4_ to recover LA, which results in the production of high quantities of CaSO_4_ as a by-product, and subsequently a lower purity solution of LA. Researchers have studied various alternative purification methods, such as membrane technology (ultrafiltration—UF, nanofiltration—NF, electrodialysis—ED), ion-exchange/adsorption, reactive distillation, and hybrid short path evaporation [2, 4, 7–9]. However, in most cases, an integrated process is required to reach high levels of purity. For instance, a downstream purification system may include a combination of different membrane separation processes (such as UF and NF), ion exchange, and vacuum-assisted evaporation [10] to reach a purity level of > 99.5%. Another, hybrid-membrane purification process that led to high-quality LA (> 90.0% purity) combined concentration, ultrasonic liquid–liquid extraction, phase separation, and distillation to recover and purify LA from the fermentation broth [11]. Although, high LA purity was achieved, the process was proven quite complex with low recovery rate, mainly due to the liquid–liquid extraction process.

LA recovery from green grass silage leachate, a by-product of green grass biorefinery, is a potentially alternative production source. Grass silage leachate is produced during grass silage maturation (fermentation), and its yield may reach up to 300–400 L per ton of fresh grass silage. LA is a major component of grass silage leachate at a typical concentration range between 22 and 38 g/L [12]. Since, a consortium of microorganisms is growing during the ensiling process both isomers D- and L-LA are present in the grass silage leachate [13, 14]. Towards recovering LA from grass silage leachate, Ecker et al. developed a LA recovery process that consisted of UF, two-step NF, ED, and reverse osmosis (RO) processes [15]. However, impurities that have a high chemical affinity to LA, such as acetic and butyric acid, remained in this LA enriched stream.

Novel biologically based removal and purification processes have gained increasing attention as potential alternatives to the conventional purification processes. Such processes may take advantage of the intrinsic high selectivity of biological catalysts (enzymes) to convert or catabolize specific impurities present in the process solution. The high selectivity is of significant importance, especially when the impurities have high chemical affinity to the main component, i.e., mixtures of short-chain organic acids or of different oligo-saccharides. Moreover, biologically based purification processes may also purify optical isomers that practically have the same physical and chemical properties; thus, their physicochemical separation can be only achieved using chiral chromatograph, that is an expensive, highly specialized method that cannot be applicable to all types of optical isomers [16]. For instance, a novel recycling process for the polylactic acid polymer (PLA), which is based on an alkaline thermochemical hydrolysis process followed by a biological purification step of the resulting lactate solution, has been suggested. Lactate solution produced by PLA hydrolyzation consists of D- and L-lactate, where L-lactate is dominant. An *E. coli* strain (DC1001) was genetically modified to selectively catabolize D-lactate without consuming L-lactate. This biologically based purification process was employed to a D- and L-LA solution and resulted in a highly purified L-LA solution (> 99%) [17, 18]. The proof-of-concept of the biological purification process was exhibited in a batch experiment of 1 L flask containing 125 g/L D- and L-LA, that resulted in almost complete (i.e., 100%) removal of D-LA after 30 h of aerobic incubation at 37 °C. The authors did not study the possibility of reusing the *E. coli* cells or different culture modes (i.e., fed-batch, semi-continuous or continuous) that would facilitate process scale-up.

Membrane Bioreactor (MBR) technology is a well-established hybrid technology in the field of wastewater treatment, advantageously combining biological processes and membrane separation in a single process step. Other advantages include the small plant footprint, high quality of effluent, efficient bacteria and viruses rejection, and stable operation even in cases of high or shock loadings [19]. MBR technology may be also employed to overcome challenges of single-strain biotechnological production (fermentation) processes, such as cells’ wash-out, removal of toxic metabolic by-products, operation in continuous mode etc. Taleghami et al. [20] studied the production of LA in a laboratory scale MBR employing *Lactobacillus bulgaricus* grown on lactose as carbon source. Both UF and NF membranes were used to separate the LA solution from the fermentation broth achieving a maximum productivity of 17.1 g L^−1^ h^−1^. MBR process has been also used for the production of bioethanol [21] and polyhydroxyalkanoates (PHA) [22] employing organic wastes or by-products as substrate. Bioethanol production through fermentation of wheat straw hydrolysate was studied using an immersed MBR and a recombinant, xylose-utilizing *Saccharomyces cerevisiae* strain achieving continuous fermentation operation and process intensification [21]. An immersed MBR was also used for the production of PHA through a semi-continuous cultivation and recovery of *Cupriavidus necator* bacteria grown on food-derived Volatile Fatty Acids (VFA) [22]. The semi-continuous mode resulted in increased bacterial tolerance to VFA concentration and maximum PHA production. Recently, Raveschot et al. [23] used *Lactobacillus helveticus* strains to produce bioactive peptides (BAP) in a continuous fermentation mode in a 3 L MBR employing a hollow-fiber microfiltration membrane. The bioreactor was continuously fed with fresh substrate at the same flow rate that the permeate was withdrawn. The integrated continuous process exhibited threefold increase of BAP productivity compared to batch fermentation allowing also the production and purification of the BAP in a single process step.

In this study, an engineered strain of *E.coli* (A1:ldhA), incapable of catabolizing LA [24], has been used to selectively catabolize and remove impurities in a synthetic medium comprising LA, glucose, acetic acid (AA), and salt (NaCl) as typical components of green grass silage leachate. Although, these impurities are not the only one found in real grass silage leachates, they are representative of the main classes of organic impurities (simple sugars and short-chain organic acids) found in such streams. The main goal is to demonstrate the technical feasibility (*proof-of-concept*) of the novel bio-purification process employing the *E.coli* (A1:ldhA) strain in an MBR with synthetic LA solutions. Therefore, it was decided that the synthetic LA solution to be rather simple and well-defined, to facilitate the concept’s development. Initially, flask tests were performed to define an optimum synthetic medium composition, and to prove the concept of the bio-purification of LA in bench-scale flask tests. Kinetic data were collected before fermentation scale-up. Then, batch fermentation was validated in bench-scale bioreactor tests, followed by semi-continuous fermentations in an MBR; the membrane performance during the MBR operation was evaluated as well, to validate the integrated MBR bio-purification process.

## Materials and methods

### Strain and media

*E.coli* (A1:ldhA), a genetically modified strain from wild type *E. coli* W3110, was used for this study provided by Dr. Joseph B. Sweeney (School of Biosystems Engineering and Food Science, University College Dublin). The strain is genetically modified to be incapable of utilizing LA, but capable to selectively consume organic acids and simple sugars. The strain stocks were stored in 25% v/v pure glycerol at -80 °C and plated on Tryptic Soy Agar (TSA) with 50 μg/mL kanamycin prior to each broth culture. A single colony was inoculated into Tryptic Soy Broth with 50 μg/mL kanamycin. Minimal M9 medium was used both at the 2nd pre-culture and the main culture. The M9 medium was composed of: 6.8 g Na_2_HPO_4_, 3.0 g KH_2_PO_4_, 1.0 g (NH_4_)_2_SO_4_, 0.5 g NaCl, 4 mL of 0.1 M MgSO_4_ × 7 H_2_O, 100 μL of 1 M CaCl_2_ × 2 H_2_O and 1 mL of SL-10 trace elements solution per liter deionized water. SL-10 solution contained 1 mL 25% v/v HCl, 1.5 g FeCl_2_ × 4 H_2_O, 70.0 mg ZnCl_2_, 100 mg MnCl_2_ × 4 H_2_O, 3.0 mg H_3_BO_3_, 190 mg CoCl_2_ × 6 H_2_O, 2.0 mg CuCl_2_ × 2 H_2_O, 24 mg NiCl_2_ × 6 H_2_O, and 36 mg Na_2_MoO_4_ × 2 H_2_O, per liter deionized water [25]. Culture media were supplemented with glucose, AA and LA (L-LA isomer) to simulate the composition of a LA solution, originating from green grass leachate after some preliminary downstream purification steps, i.e., UF for removal of suspended solids and cation exchange softening. Glucose and AA were selected as typical impurities that may be found in such process streams [12].

### Experimental procedure

#### Shake flasks experiments

A series of preliminary tests were conducted to assess the effect of the reduction to 50% and 25% of the original amount of the supplied macronutrients included in M9 minimal medium (i.e., Na_2_HPO_4_, KH_2_PO_4_, and (NH_4_)_2_SO_4_). Afterwards, the effect of different concentrations of glucose, AA, LA and NaCl on the *E. coli* strain performance in flask fermentations was assessed. First, the effect of glucose and AA concentrations was assessed for approx. 0.5, 1.0 and 2.0 g/L of glucose, and 0.5, 1.0 and 2.0 g/L of AA, respectively; the LA concentration was chosen to be approx. 5 g/L. To assess the *E. coli* strain tolerance to increasing LA concentrations batch experiments were performed, at the following LA concentrations 5, 10, 20, 30, 35 and 40 g/L, and approx. 0.5 g/L of both glucose and AA. The highest LA concentration that did not inhibit bacteria growth was selected to assess the *E. coli* strain salinity tolerance using three different NaCl concentrations (0.1, 0.5 and 1% w/v), in the presence of approx. 0.5 g/L of both glucose and AA.

In each batch experiment, a negative control flask was also prepared, comprising LA as sole carbon source, to verify that the *E. coli* strain could not catabolize LA. Each experiment lasted for 48 or 72 h with sampling every 24 h to measure the optical density at 600 nm (OD_600_), the concentration of LA, glucose and AA, and to monitor the pH. Total nitrogen (TN) and total organic carbon (TOC) were also measured to verify that no nitrogen limitation takes place and to estimate the total organic content of the fermentation medium. To complete the growth optimization in shake-flask tests, kinetic data were collected as a final step before scaling-up the process in a bench-scale bioreactor. A 12-h staggered starting time was applied to two identical fermentation batches, to obtain data during 24 h of sampling at hourly intervals (i.e., Batch 1 for the time period of 0–12 h; Batch 2 for the time period of 12–24 h).

Overnight pre-culture was performed at 37 °C and 200 rpm, in a shaking incubator (Lab Companion SI-600R). The 2nd pre-culture was incubated at 37 °C for 22 h with shaking at 200 rpm. The medium and the glassware used were sterilized in an autoclave (Raypa Steam Sterilizer) at 121 °C for 20 min. Minimal M9 media were used for shake-flask fermentation, supplemented with glucose, AA, LA, and NaCl according to the aforementioned experimental methodology. 250 mL flasks, containing 100 mL of fermentation medium, were inoculated with 4 mL (4%) inoculum from the 2nd pre-culture and were incubated at 37 °C and 200 rpm. The experiments were performed in biological triplicates.

#### Batch bioreactor fermentation tests

To gain insight in the scale-up potential of the biologically based purification process, batch cultivations were performed in a 3 L bench-scale bioreactor (BioFlo® 120, Eppendorf S.E.) with a working volume of 1.8 L. The bioreactor was inoculated with 4% v/v of the 2nd pre-culture after 22 h cultivation. Culture media contained minimal M9 medium supplemented with 0.5 g/L glucose, 0.5 g/L AA, 10 g/L LA, and 0.1% w/v NaCl. Fermentations lasted either 24 h or 30 h to achieve complete AA consumption. Temperature was set to 37 °C, agitation to 450 rpm and air flow rate to 1.7 vvm. The pH was controlled at 7.0 by automatic addition of 2.5 N NaOH and 2.5 N H_2_SO_4_ solution. Dissolved oxygen (DO) and OD_600_ were continuously monitored by the respective probes (Eppendorf Optical DO Sensor, ISM® and Hamilton Dencytee Unit 225). An hourly sampling frequency during 0–6 h and 20–28 h time periods was applied; the collected data were used to calculate growth kinetics and the efficiency of glucose and AA consumption rates, which in turn were used to design the MBR semi-continuous process.

#### Semi-continuous MBR fermentation tests

The semi-continuous MBR fermentation process comprised two stages: initially, the bioreactor was operated in batch mode that lasted from 24 to 34 h, employing the operating parameters that were tested in the batch bioreactor fermentations ("Batch bioreactor fermentation tests" section). The aim of this process stage was to increase the concentration of the active biomass in the bioreactor, while achieving almost complete catabolism/removal of both glucose and AA. Then, an intermittent removal of the treated (purified) synthetic LA solution started, followed by feeding of fresh synthetic LA solution (i.e., permeate/feed cycles). A custom-made UF membrane module [26] comprising commercial PVDF hollow fiber (0.03 μm pore size) membranes (PURONR MBR, Koch Separation Solutions) and a peristaltic pump were used to withdraw the treated (purified) synthetic solution from the bioreactor. The membrane module was submerged in the bioreactor vessel and autoclaved in-situ, since it has been certified that the membrane is not affected by the sterilization process [26]. The volumetric flow of the peristaltic pump was regulated to maintain a membrane flux of approx. 30 L/m^2^/h (LMH). The pump operated until the targeted volume of treated permeate was withdrawn through the membrane. Then, an equal volume of fresh synthetic medium was fed and fermented for different time intervals. The permeate/feed cycles were repeated achieving a semi-continuous operation of the MBR. The permeate/feed cycle time intervals and the operating conditions are summarized in Table 1.

**Table 1:** Experimental design of semi-continuous MBR experiments

		MBR 1	MBR 2	MBR 3	MBR 4	MBR 5 & 6
Overall process conditions	Nutrients in batch	25% Μ9 + TE	25% M9 + TE	25% M9 + TE	50% M9 + TE	50% M9 + TE
	Nutrients in MBR	25% Μ9 + TE	25% Μ9 + TE	50% M9 + TE	50% M9 + TE	50% M9 + TE
Batch mode	Fermentation time	24 h	30 h	30 h	24 h	34 h
Semi-continuous MBR mode						
Cycle 1	Permeate/Feed volume	300 mL	300 mL	300 mL	230 mL	230 mL
	Fermentation time	7 h	16 h	16 h	3 h	3 h
Cycle 2	Permeate/Feed volume	300 mL	200 mL	200 mL	230 mL	230 mL
	Fermentation time	7 h	8 h	8 h	3 h	3 h
Cycle 3	Permeate/Feed volume	600 mL	300 mL	300 mL	230 mL	230 mL
	Fermentation time	14 h	16 h	16 h	3 h	3 h
Cycle 4	Permeate/Feed volume	300 mL	200 mL	200 mL	230 mL	230 mL
	Fermentation time	7 h	16 h	16 h	3 h	3 h
Cycle 5	Permeate/Feed volume	-	300 mL	300 mL	-	-
	Fermentation time	-	16 h	16 h	-	-
Cycle 6	Permeate/Feed volume	-	200 mL	-	-	-
	Fermentation time	-	16 h	-	-	-

### Analytical methods

#### High-performance liquid chromatography (HPLC)

Glucose concentrations were assessed using an HPLC, Shimadzu LC-10AT VP Liquid Chromatograph (Shimadzu, Germany), using a ShodexTM Sugar SH1011 (8.0 mm I.D. × 300 mm) column and a Shimadzu Refractive Index Detector RID-10A. The same method and column were used for estimating the concentration of organic acids in the sample using a Shimadzu SPD-M20A UV detector. Fermentation broth HPLC sample preparation consisted of centrifugation at 4500 rpm for 7 min, filtration through 0.45 μm filters, and appropriate dilution. 5 mM H_2_SO_4_ was used as eluent. The flow rate was set to 0.4 mL/min, and the oven temperature to 60 °C.

#### Total organic carbon (TOC) and total nitrogen (TN)

TOC and TN were measured in a Shimadzu TOC-5000A TOC/TN Analyzer. A calibration curve of 10–100 ppm and 0.5–5 ppm were used for the TOC and TN measurements, respectively. Each fermentation broth sample was centrifuged at 4500 rpm for 7 min and diluted accordingly.

#### Optical density (OD_600_) & dry biomass

Optical density was measured for cell growth determination at 600 nm by a photometer (UV1700 Pharmaspec Shimadzu UV–Vis Spectrophotometer) during fermentation experiments in flasks. OD_600_ in bioreactor experiments were measured online through a Dencytee Arc (Hamilton Company) cell density sensor. OD_600_ data were recorded every 6 or 12 min. A correlation between OD_600_ and dry biomass was performed by regular analysis of dry biomass concentrations. This was performed using culture samples of 10 mL that were centrifuged at 4500 rpm for 7 min, the supernatant was disposed and the remaining cell-pellet was washed twice with 1 mL H_2_O. The pellet was dried at 60 °C for at least 48 h, until recording constant weight.

## Results and discussion

### Batch fermentations in shake flasks experiments

The first step towards developing the LA bio-purification process was the determination of an optimum synthetic medium composition, which will facilitate the growth of the *E. coli* strain and the subsequent experimental steps. At the same time, the synthetic medium should be representative of the composition of a typical grass silage leachate after some preliminary downstream processing. To minimize the use of macronutrients in the bio-purification process, which would reduce operational costs in large-scale applications, M9 minimal medium macronutrients concentrations were reduced to 50% and 25% of the original quantities. Results from these preliminary tests showed that the *E. coli* strain was capable of consuming both glucose and AA within 24 h under all macronutrient concentrations tested (see Additional file 1: Fig. S1). Therefore, it was decided to use 25% of the typical quantity of macronutrients included in M9 minimal media, for the subsequent experimental steps.

#### Effect of glucose and AA concentration

The *E. coli* strain growth and ability to catabolize different concentrations of glucose and AA, while leaving LA intact in the medium, was initially assessed. Results from three different shake-flask tests (Test 1, Test 2 and Test 3) with different initial concentrations of glucose and AA are presented in Table 2. The parameter of initial LA concentration was kept constant for all tests, i.e., 5 g/L, while glucose and AA concentrations were approx. 0.5 g/L in Test 1, they were doubled for Test 2 (i.e., approx. 1.0 g/L), and further increased for Test 3 (i.e., approx. 2.0 g/L). Indeed, LA concentration remained constant, roughly 5 g/L, regardless of the initial glucose and AA concentrations tested (Table 2). According to Table 2, the highest growth was recorded at 24 h for Tests 1 and 3, and at 48 h for Test 2, reaching an OD_600_ value of 0.93 ± 0.04, 0.67 ± 0.02, and 1.12 ± 0.02, respectively. At 72 h, it is obvious that all fermentations had entered stationary phase. The lowest TOC concentration within the culture medium was achieved at the end of the exponential growth phase; 2136 ppm for Test 1, 2343 ppm for Test 2, and 3228 ppm for Test 3. TN measurements showed (Table 2) that there is no nitrogen limitation until 72 h when TN was 24 ppm, 18 ppm, and 39 ppm for Tests 1, 2 and 3, respectively.

**Table 2:** Data from batch fermentation tests in shake flasks with different initial concentrations of glucose and AA

	t (h)	ΤEST 1	ΤEST 2	ΤEST 3
Glucose (g/L)	0	0.61 ± 0.03	1.24 ± 0.03	2.33 ± 0.04
	24	0.00 ± 0.00	0.00 ± 0.00	0.98 ± 0.04
	48	0.00 ± 0.00	0.00 ± 0.00	0.12 ± 0.03
	72	0.00 ± 0.00	0.00 ± 0.00	0.00 ± 0.00
AA (g/L)	0	0.54 ± 0.01	1.02 ± 0.02	2.00 ± 0.04
	24	0.36 ± 0.07	1.45 ± 0.34	2.43 ± 0.03
	48	0.09 ± 0.00	0.18 ± 0.03	2.57 ± 0.08
	72	0.00 ± 0.00	0.00 ± 0.00	2.72 ± 0.10
ΟD_600_	0	0.15 ± 0.00	0.15 ± 0.00	0.15 ± 0.01
	24	0.93 ± 0.04	0.66 ± 0.02	0.67 ± 0.02
	48	0.81 ± 0.03	1.12 ± 0.02	0.66 ± 0.01
	72	0.75 ± 0.08	0.79 ± 0.01	0.56 ± 0.01
pH	0	7.10 ± 0.00	7.10 ± 0.01	7.08 ± 0.02
	24	7.65 ± 0.10	6.59 ± 0.01	6.31 ± 0.01
	48	8.04 ± 0.05	8.52 ± 0.04	5.82 ± 0.04
	72	8.03 ± 0.04	8.89 ± 0.06	5.79 ± 0.04
LA (g/L)	0	5.21 ± 0.22	5.05 ± 0.07	4.97 ± 0.06
	24	5.25 ± 0.16	5.16 ± 0.04	5.06 ± 0.05
	48	5.15 ± 0.14	5.12 ± 0.08	5.13 ± 0.14
	72	5.15 ± 0.11	5.21 ± 0.10	5.18 ± 0.05
TN (ppm)	0	62	62	63
	24	32	49	45
	48	31	24	48
	72	24	18	39
TOC (ppm)	0	2748	3051	3804
	24	2136	2693	3228
	48	2303	2343	3820
	72	2186	2405	3596

**Fig. 1:**
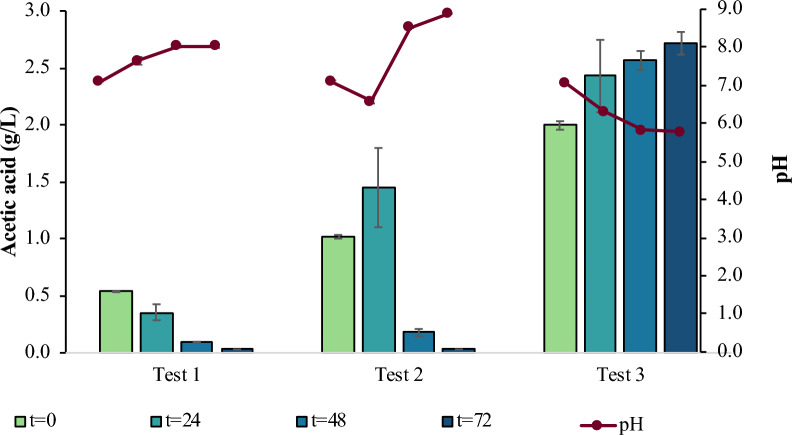
Results from batch fermentation tests in shake flasks with different concentrations of glucose and AA; AA concentration at different fermentation time points compared to fermentation broth pH profile; Test 1: 0.5 g/L glucose + 0.5 g/L AA; Test 2: 1.0 g/L glucose + 1.0 g/L AA; Test 3: 2.0 g/L glucose + 2.0 g/L AA

The concentration of glucose and AA in the synthetic LA solution was analyzed to assess their removal efficiency. Initial glucose concentrations were slightly higher than the targeted values, which did not seem to affect the experimental procedure. Complete glucose consumption was observed at 48 h for Test 3 (2.33 ± 0.04 g/L initial glucose concentration), while glucose was consumed within 24 h for Test 1 (0.61 ± 0.03 initial glucose concentration) and Test 2 (1.24 ± 0.03 initial glucose concentration). Due to glucose consumption, AA was excreted in the culture media as described previously [27]; in fact, the higher the initial glucose concentration the higher the AA production. Similar behavior was observed in Test 3; the *E. coli* cells first consumed glucose inhibiting the consumption of less preferred carbon sources such as AA, following a classic diauxic growth pattern. In addition, acetate overflow was also observed, meaning that part of the uptaken glucose into *E. coli* cells was first catabolized into AA and excreted into the medium. According to the literature, the excreted AA can be used by *E. coli* for cell growth after glucose consumption, named “acetate switch” [28]. However, in case of Test 3, the production of AA reduced media pH, possibly hindering further bacteria growth (Fig. 1). In particular, during Test 3, AA increased from 2.00 ± 0.04 g/L to 2.72 ± 0.10 g/L and pH decreased from 7.08 ± 0.02 down to 5.79 ± 0.04 within 72 h, whereas for Test 2, the concentration of AA increased roughly by 0.50 g/L, while pH decreased to 6.59 ± 0.01 at t = 24 h. Afterwards, AA started to be consumed, which resulted to a pH increase from 6.59 ± 0.01 to 8.89 ± 0.06 at t = 72 h [29]. As growth under higher glucose concentrations was hindered likely due to the overflow metabolism of glucose, which results in AA excretion and the corresponding pH reduction, the initial concentration of 0.5 g/L for both glucose and AA was chosen as the optimum for further experimental set ups.

#### Effect of LA concentration

**Fig. 2:**
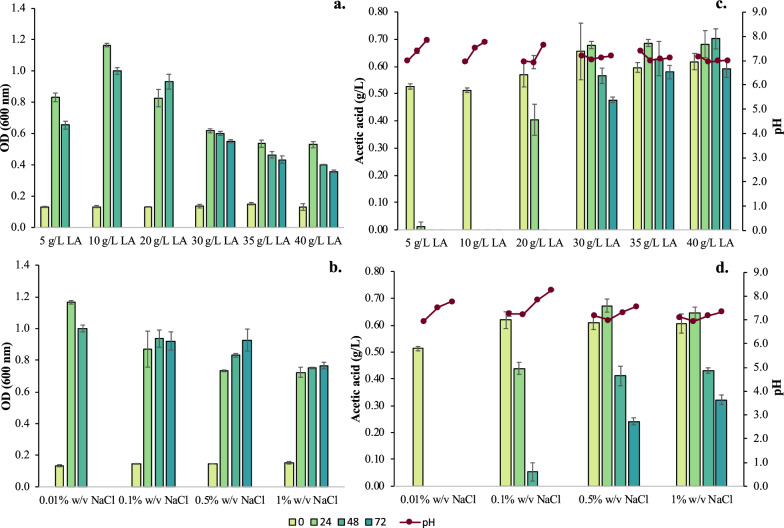
Results from batch fermentation tests of 72 h in shake flasks with different concentrations of LA and NaCl. **a** Growth of *E.coli* cells in the presence of different LA concentrations; **b** OD_600_ of *E.coli* cells in the presence of 10 g/L LA and for different concentrations of LA; **c** AA concentration in the fermentation broth compared to broth’s pH profile different NaCl concentrations; **d** AA concentration in the fermentation broth compared to broth’s pH profile in the presence of 10 g/L LA and different NaCl concentrations

The second parameter that was assessed, for its effect on the *E. coli* strain growth, was LA concentration. Six different LA concentrations of 5, 10, 20, 30, 35 and 40 g/L were tested. As illustrated in Fig. 2a, cell growth was lower for concentrations above 20 g/L. In particular, for 30 g/L LA, cells reached an OD_600_ of 0.62 ± 0.01, while for 35 and 40 g/L LA, a value of 0.54 ± 0.02 and 0.53 ± 0.02 was achieved, respectively. However, for 10 g/L LA, a maximum OD_600_ value of OD_600_ = 1.17 ± 0.01 was observed at 24 h. The maximum OD_600_ measurement that was observed at 48 h for the *E. coli* strain grown on 20 g/L LA, was comparable to that at 24 h for 5 and 10 g/L of LA. Surprisingly, glucose was consumed within 24 h in all fermentations. On the other hand, the AA consumption seems to be affected, as depicted in Fig. 2b. In the presence of 5 and 10 g/L LA, AA was consumed within 24 h. In the case of 20 g/L LA, AA consumption was delayed and was totally consumed within 48 h, while for all LA concentrations above 20 g/L, AA was not totally consumed, even at longer fermentation times (i.e., 72 h). LA concentration remained constant during all the fermentations, which is very encouraging as it reflects the selective catabolism of other organic compounds under all experimental conditions. Considering that the rate of glucose and AA consumption is significant for the process performance and that the *E. coli* strain performance declined for LA concentrations above 10 g/L, an LA concentration of 10 g/L was selected for all future experiments.

#### Effect of NaCl concentration

The last set of experiments focused on the impact that salinity has on the *E. coli* strain performance. Salinity tolerance (i.e., NaCl concentration) was assessed by testing three different concentrations of salt in the synthetic medium, i.e., 0.1, 0.5 and 1% w/v NaCl. Typically, M9 media and accordingly the synthetic media in the previous experiments were characterized by 0.01% w/v salinity, while in grass silage NaCl concentration may reach up to 10 g/L (i.e., approx. 1.0% w/v) NaCl [30]. The flask fermentation process was negatively affected for increased NaCl concentrations (i.e., 0.1, 0.5 and 1.0% w/v), but the effect was more prominent for 0.5% and 1% w/v NaCl (Fig. 2c). For 0.1% w/v NaCl, an OD_600_ of 0.94 ± 0.05 at t = 48 h was observed, while for 1% w/v NaCl, an OD_600_ of 0.75 ± 0.00 at t = 48 h. In addition, Fig. 2d shows that 100% removal of AA was achieved in the presence of 0.1% w/v NaCl, whereas only 60.7% and 47% of AA was consumed within 72 h, when 0.5% w/v and 1% w/v NaCl was added to the synthetic media, respectively. Glucose consumption was not affected as NaCl concentration increased; glucose was totally depleted within 24 h. On the contrary, pH values were affected mainly due to the different AA consumption profiles. At 0.01 and 0.1% w/v NaCl, the AA was totally consumed at t = 72 h and the pH values rose around 8.0. At higher NaCl concentrations, due to the incomplete AA consumption the pH was maintained around 7.0 which is the optimum value for the *E. coli* cells to grow.

In summary, the synthetic medium simulating the composition of grass silage leachate used in all the subsequent experiments comprised 0.5 g/L glucose and 0.5 g/L AA as carbon sources, 10 g/L LA and 0.1% w/v NaCl. Furthermore, the series of experiments for defining the composition of the synthetic medium that does not affect the *E. coli* strain’s growth, offered preliminary evidence that the strain can selectively catabolize organic impurities (i.e., glucose and AA) in the presence of different concentrations of LA, without catabolizing any of the LA present. Kinetic data collection under these conditions followed to design the bench-scale batch bioreactor experiments.

### Kinetic data collection in shake flask experiments

**Fig. 3:**
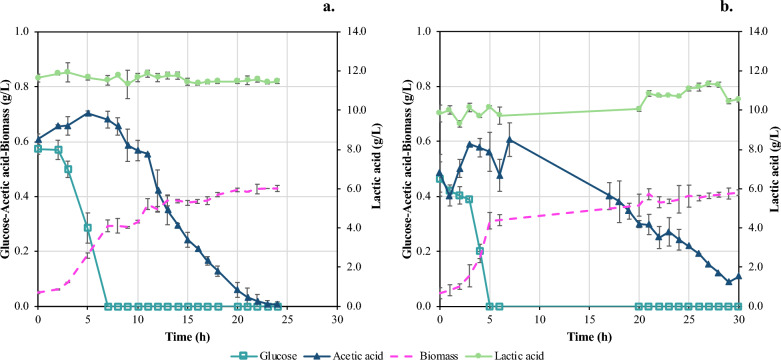
Comparison of results from batch fermentation test in shake flasks and bench scale bioreactor for kinetic data collection. The synthetic solution comprises: 0.5 g/L AA, 0.5 g/L glucose, 10 g/L LA and 0.1% w/v NaCl. Temporal profile of glucose, AA and cells’ growth (left *y*-axis) and LA concentration (right *y*-axis); **a** Batch fermentation in flask-scale; **b** Batch fermentation in 3 L bench-scale bioreactor

The last step in the flask scale experiments was the collection of kinetic data on the *E. coli* growth and the removal of glucose and AA. These results would provide valuable data for the design of the up-scaled bioreactor fermentation process. Two different batch tests were performed by 12-h time difference, and samples were collected on an hourly base to cover a total period of 24 h. Figure 3a shows that glucose was consumed within 7 h, while LA remained constant at 11.69 ± 0.33 g/L. AA was produced by the cells in the first 7 h reaching 0.70 ± 0.01 g/L, and was totally consumed within 24 h, afterwards. The cells started using AA as carbon source only after complete consumption of the glucose [28]. This is a typical diauxic growth behavior, which has been widely reported in the relevant literature [31, 32].

According to Fig. 3a*, E. coli* cells present a short lag phase and grow exponentially from 3 to 7 h of fermentation while glucose was catabolized by the cells, reaching 0.29 ± 0.02 g/L concentration of dry biomass. The cells then grew at a slower rate when AA was present in the culture as sole carbon source. The final biomass concentration at 24 h of fermentation reached up to 0.43 g/L concentration of dry biomass. pH was also measured during the kinetic study. At t = 0, pH was 7.11 ± 0.00, and subsequently dropped down to 6.80 ± 0.02 during glucose consumption and AA excretion. From 8 to 24 h, pH increased up to 7.10 ± 0.02, which is near the optimum value for *E. coli* growth [33].

### Batch bioreactor fermentation tests

Batch fermentation experiments in the lab-scale bioreactor were designed according to the aforementioned kinetic data results. The duration in batch bioreactor fermentation tests was prolonged for 6 h, since AA consumption was not complete after 24 h (Fig. 3b). Biomass growth was similar for flasks and bioreactor experiments, reaching at 24 h of fermentation up to 0.43 ± 0.01 g/L and 0.39 ± 0.03 g/L, respectively. However, comparing the growth curves in flasks and bioreactor fermentations, a slight difference is observed concerning the lag- and the exponential-phase. Fermentation in flasks exhibited a longer lag-phase which lasted for 3 h, while in the bioreactor cells grew faster, probably due to better aeration and mixing conditions of the bioreactor. Subsequently, cells enter a stationary phase with 2-h difference, at t = 7 h for flasks and at t = 5 h for bioreactor tests, reaching similar concentrations of biomass (Fig. 3b). Exponential growth ended, when glucose has been totally depleted in both cases. LA concentration remains constant in bioreactor experiments just like shake flask tests. Regarding AA concentration in bioreactor tests, a similar trend to flasks tests is observed, i.e., excretion of AA while glucose is consumed. However, AA is not totally consumed in bioreactor tests, given that at 20 h of fermentation its concentration is 0.30 g/L ± 0.01. AA consumption rate was calculated to assess the efficiency of the process in bioreactor tests compared to flask tests. AA consumption rate is rather linear both in flasks and bioreactor tests, as depicted in Fig. 3. However, the AA consumption rate in flasks is higher (0.035 g/L/h) compared to the bioreactor (0.022 g/L/h) resulting in 60.4% removal in the bioreactor within 30 h compared to 100% AA removal in flask within 24 h. These results could be explained due to the challenges associated to fermentation scale-up, such as non-uniforms conditions in the bioreactor that may cause negative effects on the strain or different physiological responses to high shear-flow dynamics, gas–liquid mass transfer limitations, substrate concentration gradients etc. [34]. The results from the batch fermentations in the bench-scale bioreactor were quite promising since the *E. coli* strain could successfully catabolize the organic impurities (i.e., glucose and AA), while LA concentration remained constant. Summarizing all the above results, it can be stated that the development of an LA bio-purification process using the *E. coli* strain seems to be feasible and provide a valid proof-of-concept that could be further developed to a semi-continuous MBR process.

### Semi-continuous MBR fermentation tests

The concept of the LA bio-purification was further studied by testing different operating parameters in a semi-continuous MBR process aiming to provide a clear *proof-of-concept* of the integrated bio-purification system. The semi-continuous process was divided in two stages, the initial fermentation process (1st stage), which was similar to the batch fermentation described in "Batch bioreactor fermentation tests" section, and the 2nd stage when cycles of membrane filtration operation and biological fermentation process (i.e., the MBR concept) are repeated to provide a purified LA effluent. The 1st stage aims to develop a mature cell culture in addition to the total depletion of glucose and AA contained in the initial fermentation broth, before adding fresh fermentation medium for further cells’ growth.

**Fig. 4:**
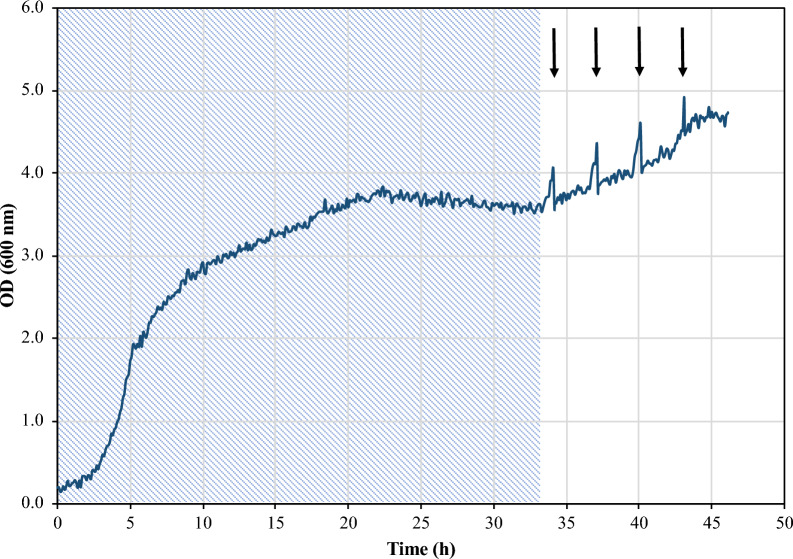
Cells’ growth profile in MBR 6 test obtained by online monitoring of OD_600_; shaded area corresponds to the batch operation of the MBR (1st stage) while arrows indicate the time points of the permeate/feeding cycles during the semi-continuous MBR operation

The growth behavior for all the MBR experiments (6 in total), during the 1st stage of the process was similar, while the cells’ growth differs depending on the applied conditions of each MBR test, when the process entered in the semi-continuous MBR mode (i.e., 2nd stage), (Additional file 1: Fig. S2). A typical OD_600_ profile is depicted in Fig. 4, concerning MBR 6 tests. An exponential growth is observed for the first 5 h which concurs with the glucose removal by the cells. By the end of the exponential growth, OD_600_ is approx. 2.0. Afterwards, AA is used as the sole carbon source and the cells kept growing at a slower rate and by the end of the batch operating stage the cells have reached stationary phase. When the periodic permeate/feeding cycles start, distinctive peaks appear during OD_600_ monitoring, which correspond to the intermittent removal of the treated (purified) synthetic LA solution through the UF membrane module. This is clear evidence that cells are retained by the UF membrane module, while the permeate (i.e., treated LA solution) is withdrawn. Since the volume of the fermentation broth is reduced, while the cells are retained within the bioreactor, the cells concentration increases. When fresh synthetic medium is added in the bioreactor, the fermentation broth volume is restored to its initial value and a rather sharp drop of OD_600_ is observed. Afterwards, cells continue growing using the carbon sources of the fresh media.

As in flask and batch-bioreactor experiments, the AA removal determines the process design of the MBR system, according to obtained data. *E. coli* cells initially consume glucose quite fast and then AA at a slower rate, following the diauxic growth pattern, as it has already been mentioned. Glucose was consumed in less than 1 h, after each feeding step with fresh synthetic medium; however, AA removal was slower and, in some cases, not consumed at all. In Table 3, data of critical metrics referring to the semi-continuous MBR tests are provided. It is important to mention that concerning LA, it remained constant at the initial concentration with minor variation.

**Table 3:** Overview of MBR tests and calculated metrics for the efficiency of the MBR process

Test No	Semi-continuous operation time (h)	AA max consumption rate (g/L/h)	Mean LA (g/L)	Net permeate rate (mL/h)	TMP max. drop (mbar)
MBR 1	87	0.0061	10.2 ± 0.8	11.30	− 72
MBR 2	72	0.0206	10.9 ± 0.9	25.27	− 37
MBR 3	64	0.0202	11.9 ± 0.4	17.11	− 38
MBR 4	12	0.0376	11.9 ± 0.3	66.67	− 32
MBR 5	12	0.0426	11.0 ± 0.2	76.67	− 29
MBR 6	12	0.0764	11.0 ± 0.8	76.67	− 39

Table 3 shows that AA maximum consumption rate was extremely low in experiment MBR 1, which actually means that AA was practically not removed. AA maximum consumption rate corresponds to the highest value of the AA consumption rate calculated separately for each filtration/feed cycle of the respective MBR test. In MBR 2 experiment, more time (i.e., 30 h instead of 24 h) was provided to the cell culture for the 1st stage of the process to achieve total depletion of AA, before starting the 2nd stage of the semi-continuous process. It was assumed that this would improve the 2nd stage of the process, and as a result the efficiency of the entire MBR process, would be improved. Indeed, AA consumption rate increased from 0.0061 g/L/h to 0.0206 g/L/h, but still remained low in comparison with the previous flask and batch experimental steps. The total removal of AA in MBR 2 was 68.8% and the permeate recovery rate was increased from 11.30 mL/h to 25.27 mL/h; however, the process duration remained quite long.

A possible explanation may be the insufficient N concentration that was observed both at the start, as well as at the end of each feeding cycle (i.e., 7.0 ± 3.0. and 6.0 ± 0.4 in average for MBR 1 and 2 tests, respectively). To solve this issue, the M9 quantity, for the 2nd stage of the process, was doubled in MBR 3. However, the efficiency of the process was still rather low. Although, AA depletion reached 100%, the AA maximum consumption rate remained almost the same, possibly also due to the long-time intervals for each membrane filtration/fermentation cycle. When nitrogen concentration was doubled for the whole process in MBR 4 test, the AA maximum consumption rate reached 0.0376 g/L/h and the net permeate recovery was more than 3 times higher (i.e., 66.67 mL/h) compared to MBR 3. However, the AA was not totally catabolized during the batch process (1st stage) within 24 h; possibly, more time was necessary for the cells to remove the initial AA quantity.

Therefore, in MBR 5 test, the batch operation (1st stage) of the MBR process was increased by 10 h (34 h in total), to achieve complete depletion of the available carbon sources (both glucose and AA) by the cells, before starting the membrane filtration. Thereafter, an effluent, free of impurities (i.e., AA, glucose and cells) was achieved in MBR 5 within a reasonable timeframe. i.e., 3 h permeate/feeding cycle for a permeate/feeding volume of 230 ml, which is approx. 12.5% of the MBR working volume. The final optimized operating protocol was repeated in MBR 6 for validation purpose. Permeate recovery rate was identical for MBR 5 and 6 (i.e., 76.67 mL/h) and it was increased by 10 mL/h compared to MBR 4. It was also obvious that, the hindering of cell’s catabolism due to lack of nitrogen availability had been solved; the mean TN concentration at the end of each feeding cycle was approx. 50–60 ppm. Finally, the max consumption rate of AA was 0.0764 g/L/h for the optimum MBR working conditions (MBR 6).

The transmembrane pressure (TMP) of the UF filtration in the MBR is another operating parameter of high importance, which was monitored and assessed in this study. The membrane filtration performance was evaluated by measuring TMP which indicates the level of the filtration resistance due to membrane fouling. Table 3 summarizes critical parameters related to the TMP measurements for the semi-continuous MBR process. It is shown that, the maximum TMP value varied between − 29 and − 72 mbar. The overall TMP drop is rather low and fouling does not seem to be an issue for this process, possibly due to the low cell culture density and the limited production of extracellular polymers by this *E. coli* strain.

**Fig. 5:**
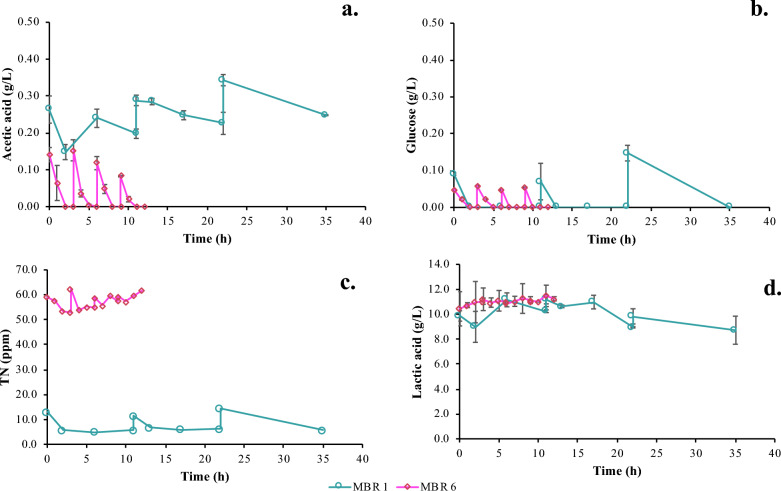
Concentration profiles of AA, Glucose, TN and LA of the initial semi-continuous MBR test (MBR 1) and the final optimized MBR test (MBR 6); Time refers to the 2nd stage of the integrated MBR process; **a** AA concentration profile; **b** Glucose concentration profile; **c** TN concentration profile; **d** LA concentration profile

Figure 5 provides a comparison between the 1st semi-continuous MBR experiment (MBR 1) with the final optimized process (MBR 6). The presented data refer to the 2nd stage of the process (i.e., filtration/feeding cycles). Thus, timepoint 0 corresponds to the point of the 1st fresh synthetic medium feed. It is evident that the duration of the process regarding the MBR operation (2nd stage), has been significantly reduced from 35 to 12 h. TN measurements also depicted in Fig. 5 clearly show that the low N concentration in MBR 1 (i.e., below 13 ppm) affects the efficiency of the process compared to the MBR 6 in which sufficient N (above 53 ppm) is provided. In both cases, glucose was consumed and LA remained constant; however, AA removal is 12.5 times higher for the optimized process (MBR 6) when compared to the initial trial (MBR 1). The 100% AA depletion before each cycle of the membrane filtration coupled with 6.8-fold higher permeate recovery rate, reflects positively on the performance of the integrated MBR process.

**Fig. 6:**
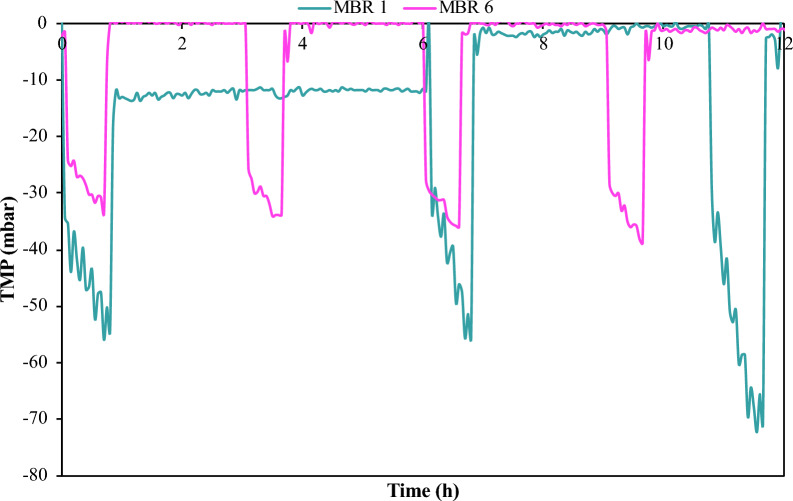
Temporal variation of TMP during membrane filtration cycles for the initial MBR test (MBR 1) and the final optimized MBR test (MBR 6)

Table 4 provides data regarding the membrane performance for the filtration cycles of two MBR experiments, namely MBR 1 (initial) and MBR 6 (optimized). It is observed that the maximum reduction in TMP occurs in the 3rd filtration cycle for both MBR 1 (-70.8 mbar) and MBR 6 (-36.0 mbar). According to the membrane specifications, the maximum operating filtration TMP drop is 600 mbar [35]. Therefore, even if the maximum TMP reduction is double for MBR 1, compared to MBR 6, the fouling of the membrane is minimum in both experiments. Lower TMP drop rate has been also achieved after process optimization, e.g., -48.6 mbar/h compared to -15.1 mbar/h at the 3rd membrane filtration cycle of MBR 1 and 6, respectively. The membrane fouling tendency was further evaluated through the TMP temporal variation, during the filtration cycles of MBR 1 and MBR 6. The overall efficient membrane filtration performance for both is evident from Fig. 6.

**Table 4:** Calculated parameters for the comparison of the membrane performance of the initial test in MBR (MBR 1) and the final optimized MBR test (MBR 6)

Test No	Filtration Cycle	Max ΤΜP drop (mbar)	Linear TMP drop rate (mbar/h)
MBR 1	1st	− 54.5	− 23.9
	2nd	− 55.7	− 38.6
	3rd	− 70.8	− 48.6
MBR 6	1st	− 33.6	− 14.5
	2nd	− 33.9	− 14.4
	3rd	− 36.0	− 15.1

## Conclusions

An optimized synthetic medium was determined, for the growth and study of an engineered *E. coli* strain capable of selectively catabolizing different carbon sources present in a synthetic LA solution. Its cultivation and the kinetics of glucose and AA were validated in bench-scale bioreactor followed by the semi-continuous MBR process experiments. Complete cell retention without significant membrane fouling and a cell-free effluent with higher purity of LA product-solution were obtained, due to 100% removal of typical impurities (i.e., glucose and AA) in short to medium operating time.

To conclude, it was clearly demonstrated that a bio-purification process for LA employing the engineered *E. coli* strain cultivated in an MBR process is a technically feasible concept, while the advantages of an efficient semi-continuous MBR bio-purification process are obvious and the technology is worth to be further investigated and scaled-up. Further research towards the validation of the bio-purification process should be performed with real LA fermentation solutions. Furthermore, improved *E. coli* strains that could tolerate higher LA and/or salts concentrations can be developed either through classic adaptive laboratory evolution (ALE) or genetic engineering approaches, to expand the operating conditions of the bio-purification process.

### Supplementary Information

**Additional file1: Figure S1**. Results from batch fermentation tests (t = 72 h) in shake flasks with 0.5 g/L glucose, 0.5 g/L AA, 5 g/L LA and different quantities of nutrients; **a** OD_600_ of *E. coli* cells; **b** AA concentration; and **c** Glucose concentration at different time points. **Figure S2**. Cells’ growth profile in semi-continuous MBR fermentation tests MBR 1 to MBR 6 obtained by online monitoring of OD_600_.

## Data Availability

All data generated during this study are included in this published article and its supplementary information files.
