# Supplementary material for: Development of a hybrid bio-purification process of lactic acid solutions employing an engineered E. coli strain in a membrane bioreactor
Source: Biotechnol Biofuels Bioprod. 2024 Mar 30;17:48. doi: 10.1186/s13068-024-02497-2 (PMC10981347; doi:10.1186/s13068-024-02497-2)
Supplement: Supplementary file 1 — Additional file1: Figure S1. Results from batch fermentation tests (t = 72 h) in shake flasks with 0.5 g/L glucose, 0.5 g/L AA, 5 g/L LA and different quantities of nutrients; a OD600 of E. coli cells; b AA concentration; and c Glucose concentration at different time points. Figure S2. Cells’ growth profile in semi-continuous MBR fermentation tests MBR 1 to MBR 6 obtained by online monitoring of OD600. [file 13068_2024_2497_MOESM1_ESM.pdf]

Development of a hybrid bio-purification process of lactic acid solutions employing an engineered *E. coli* strain in a membrane bioreactor

Alexandra Nastouli [1,2], Joseph Sweeney [3], Michael Harasek [2], Anastasios J. Karabelas [1] and Sotiris I. Patsios[1,*]

[1] Laboratory of Natural Resources and Renewable Energies, Chemical Process & Energy Resources Institute (CPERI), Centre for Research and Technology-Hellas (CERTH), Thermi, Thessaloniki, Greece; a.nastouli@certh.gr (A.N.); karabaj@certh.gr (AJK); patsios@certh.gr (S.I.P.)

[2] Institute of Chemical, Environmental and Bioscience Engineering, TU Wien, Vienna, Austria; michael.harasek@tuwien.ac.at

[3] School of Biosystems and Food Engineering, University College Dublin (UCD), Belfield, Dublin, Ireland; joseph.sweeney@ucd.ie

*Correspondence: patsios@certh.gr; Tel.: +30 2310498183

Supplementary figures:

Figure S1. Results from batch fermentation tests (t = 72 h) in shake flasks with 0.5 g/L glucose, 0.5 g/L AA, 5 g/L LA and different quantities of nutrients; a. OD_{600} of E.coli cells; b. AA concentration; and c. Glucose concentration at different time points.

Figure S2. Cells' growth profile in semi-continuous MBR fermentation tests MBR 1 to MBR 6 obtained by online monitoring of OD_{600}.

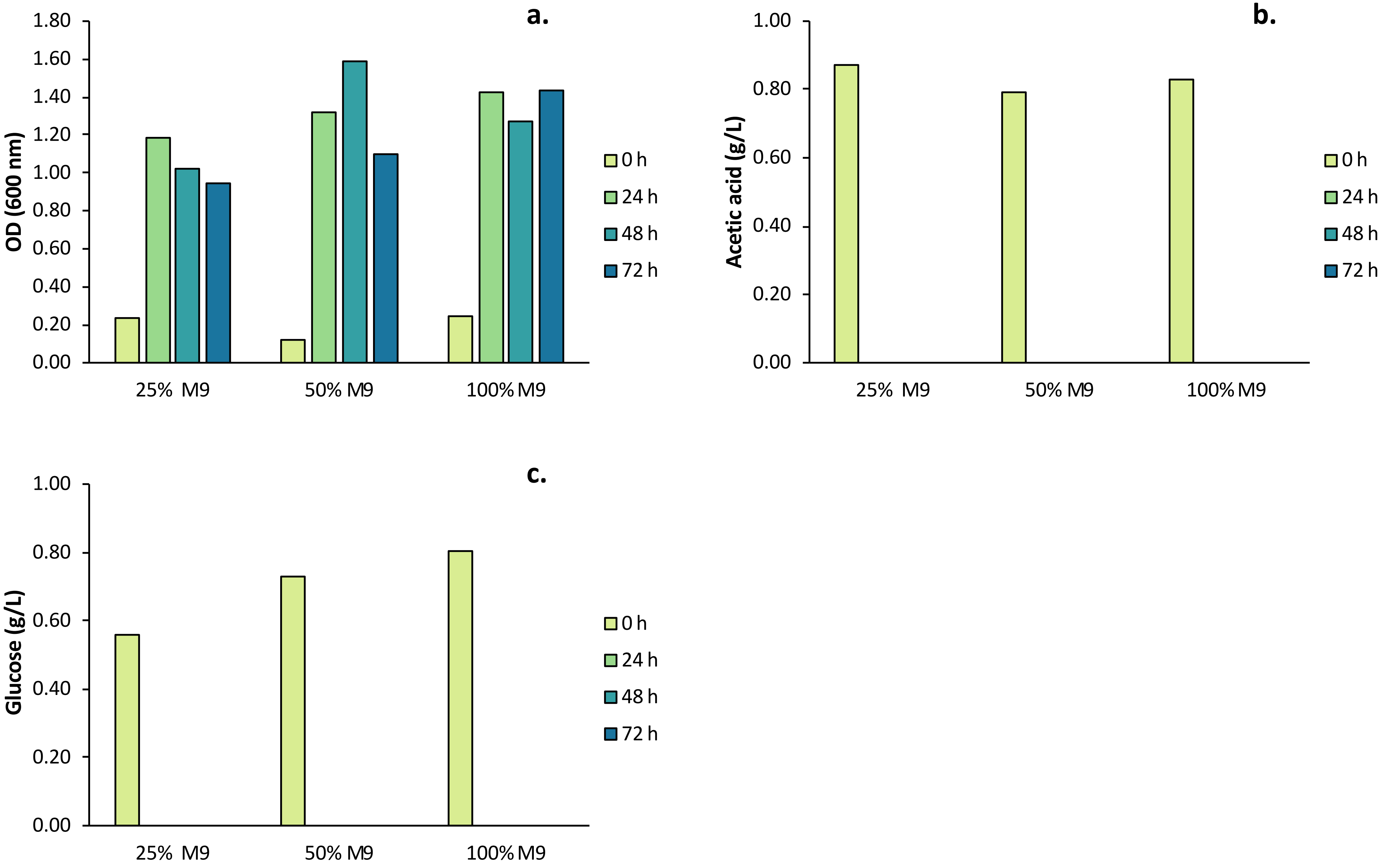

Figure S1. Results from batch fermentation tests (t = 72 h) in shake flasks with 0.5 g/L glucose, 0.5 g/L AA, 5 g/L LA and different quantities of nutrients; a. OD_{600} of E.coli cells; b. AA concentration; and c. Glucose concentration at different time points.

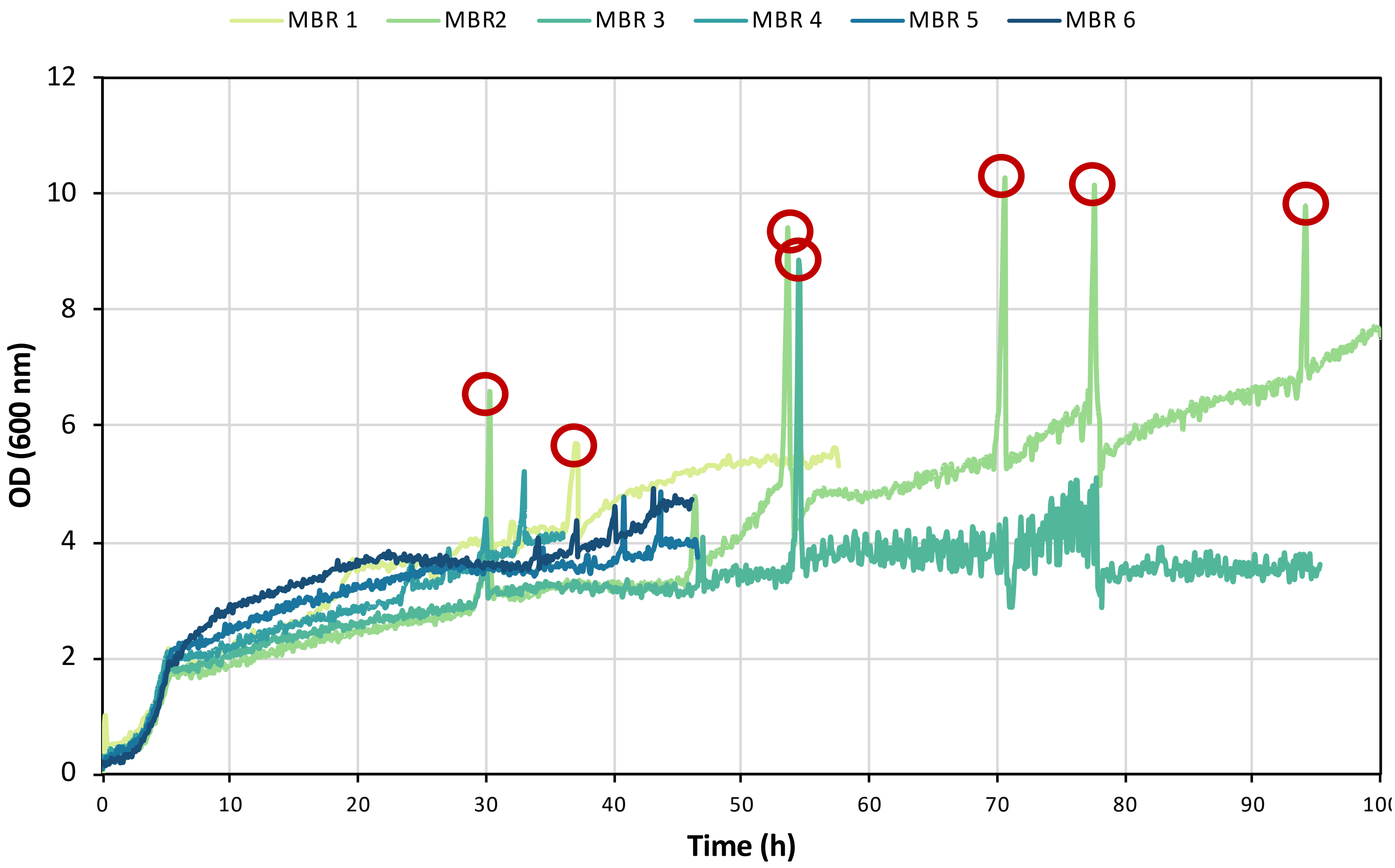

Figure S2. Cells' growth profile in semi-continuous MBR fermentation tests MBR 1 to MBR 6 obtained by online monitoring of OD_{600}.
